# Conserved associations between G-quadruplex-forming DNA motifs and virulence gene families in malaria parasites

**DOI:** 10.1186/s12864-020-6625-x

**Published:** 2020-03-17

**Authors:** Hunter L. Gage, Catherine J. Merrick

**Affiliations:** Department of Pathology, Cambridge University, Tennis Court Road, Cambridge, CB2 1QP UK

**Keywords:** *Plasmodium*, Laverania, Malaria, G quadruplex, *var* genes, *pir* genes

## Abstract

**Background:**

The *Plasmodium* genus of malaria parasites encodes several families of antigen-encoding genes. These genes tend to be hyper-variable, highly recombinogenic and variantly expressed. The best-characterized family is the *var* genes, exclusively found in the Laveranian subgenus of malaria parasites infecting humans and great apes. *Var* genes encode major virulence factors involved in immune evasion and the maintenance of chronic infections. In the human parasite *P. falciparum*, *var* gene recombination and diversification appear to be promoted by G-quadruplex (G4) DNA motifs, which are strongly associated with *var* genes in *P. falciparum*. Here, we investigated how this association might have evolved across *Plasmodium* species – both Laverania and also more distantly related species which lack *var*s but encode other, more ancient variant gene families.

**Results:**

The association between *var* genes and G4-forming motifs was conserved across Laverania, spanning ~ 1 million years of evolutionary time, with suggestive evidence for evolution of the association occurring within this subgenus. In rodent malaria species, G4-forming motifs were somewhat associated with *pir* genes, but this was not conserved in the Laverania, nor did we find a strong association of these motifs with any gene family in a second outgroup of avian malaria parasites. Secondly, we compared two different G4 prediction algorithms in their performance on extremely A/T-rich *Plasmodium* genomes, and also compared these predictions with experimental data from G4-seq, a DNA sequencing method for identifying G4-forming motifs. We found a surprising lack of concordance between the two algorithms and also between the algorithms and G4-seq data.

**Conclusions:**

G4-forming motifs are uniquely strongly associated with *Plasmodium var* genes, suggesting a particular role for G4s in recombination and diversification of these genes. Secondly, in the A/T-rich genomes of *Plasmodium* species, the choice of prediction algorithm may be particularly influential when studying G4s in these important protozoan pathogens.

## Background

Malaria is caused by protozoan *Plasmodium* parasites: in humans it causes considerable morbidity and is still responsible for almost half a million deaths each year [1]. Most severe cases of human malaria are caused by *Plasmodium falciparum*, but a further five parasite species can infect humans and there are many more species that infect rodents, birds and other vertebrates. In all vertebrate hosts, the disease involves cyclical infection of erythrocytes by *Plasmodium* parasites. The infected cells are exposed to circulating immune factors and also to splenic clearance, yet many malarias can lead to both chronic and repeated infections, indicating that the parasites have considerable capacities for immune evasion. These capacities have been linked, in several species, to the variant expression of virulence factors that are exposed on the infected erythrocyte surface and are encoded by highly variable families of variantly-expressed virulence genes.

**Fig. 1:**
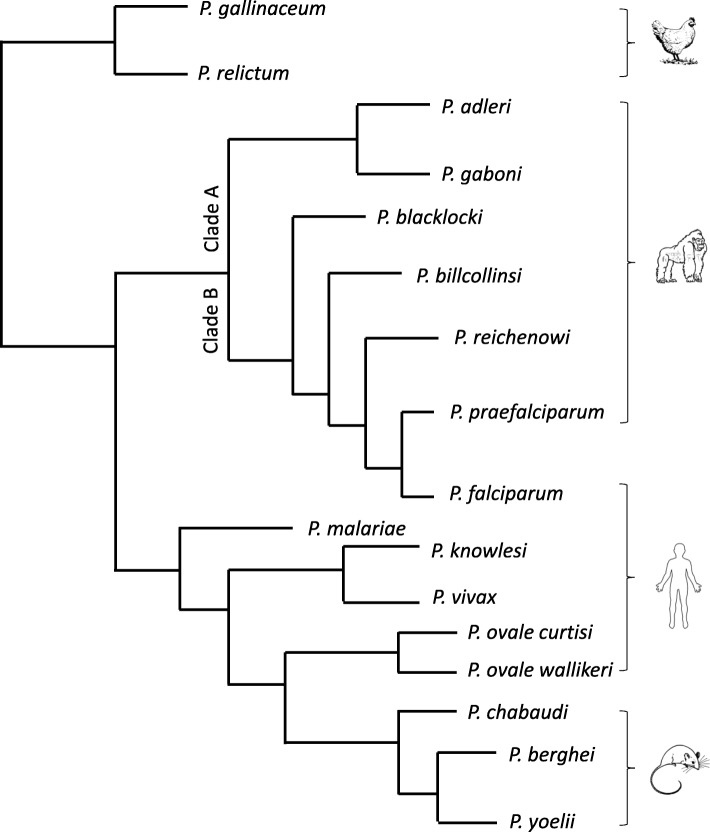
Phylogenetic tree of Laveranian, rodent, avian and human-infecting *Plasmodium* parasites. A phylogenetic tree depicting evolutionary relationships among *Plasmodium* parasites was compiled from existing published data [10, 11]. The tree is a schematic representation; thus, branch lengths do not reflect true evolutionary distances

Such gene families are best-characterized in *P. falciparum*, where the *var* gene family encodes ~ 60 variants of *P. falciparum* Erythrocyte Membrane Protein 1 (PfEMP1) [2–4], an adhesin which allows infected erythrocytes to adhere to the vascular endothelium and avoid splenic clearance. PfEMP1 proteins are critical virulence factors and they also contribute to disease, exacerbating vascular occlusion, hypoxia and lethal syndromes such as cerebral malaria. *Var* genes are restricted to the Laveranian subgenus of *Plasmodium*, which infects great apes and includes the human-infecting *P. falciparum* species [5], but other *Plasmodium* species encode other gene families that may have similar roles, including the *sicavar* family in the macaque parasite *P. knowlesi* [6, 7] and the *pir* family which appears widely in many species from rodent to human malarias [8, 9] (Fig. 1).

Effective antigenic variation over long periods of time requires highly regulated and mutually exclusive gene expression, and this has indeed been well characterized in the case of the *var* genes [12]. Other families, including *sicavar* [13] and *pir* [14], appear to have similar dynamics, albeit with less tight mutually-exclusive regulation. In the case of *P. falciparum*, *var* genes are regulated by epigenetic silencing and expression switching [12], and additional antigenic diversity is generated within the ~ 60-member gene family via frequent recombination during both mitosis and meiosis [15, 16]. Thus, *var* gene regulation is a key virulence mechanism for the maintenance of chronic infections caused by *P. falciparum*: understanding this at the molecular level is an area of great interest in malaria biology.

A decade ago, it was first observed that many *var* genes are associated with DNA motifs that could form G-quadruplexes (G4s) [17]. G4s are DNA or RNA secondary structures of the general form *G*_3_*N*_(0 − 11)_*G*_3_*N*_(0 − 11)_*G*_3_*N*_(0 − 11)_*G*_3_. They are composed of G-quartets, planar arrangements formed from Hoogsteen hydrogen bonding between four guanines, which stack on top of one another to form four-stranded, non-helical structures. Each G-quartet is typically stabilized by a monovalent cation (Na^+^ or K^+^), and short loops of other nucleotides run between them. G4s can form intra- or intermolecularly, and can involve one, two or four oligonucleotide strands, but the ‘simplest’ intramolecular form is the best studied and its ability to fold both in vitro and in vivo has been shown in human cells [18]. G4s distort the double-helical structure of DNA, and can play important roles in the regulation of gene transcription [19], DNA replication [20] and telomere maintenance [21] in eukaryotes. Their potential role in *var* gene biology is of clear interest, particularly because *Plasmodium* genomes tend to be extremely G/C-poor [22] and accordingly have a striking paucity of G-rich G4-forming motifs. This, in turn, strongly suggests that there is a selective advantage for conserving G4 motifs that appear together with *var* genes.

Investigations conducted both in silico and in cultured parasites have recently established that G4-forming motifs indeed have a probable role in recombination and evolution of *var* genes in *P. falciparum*. The motifs strongly associate with recombination breakpoints that were observed in *var* genes when parasites were cultured over many mitotic cycles, evolving new gene variants [23]. Furthermore, the knockdown of a RecQ helicase homolog called *Pf*WRN dramatically increased the rate of *var* gene recombination, as would be expected if this helicase was responsible for unwinding G4s and modulating their recombinogenic effects [24].

Here, we set out to examine in detail how the association between G4s and virulence genes might have evolved across the *Plasmodium* genus, and the extent to which the association is conserved among *Plasmodium* species. Our previous work had suggested that the association is not unique to *P. falciparum*: it also appeared in a second, closely-related Laveranian genome, *P. reichenowi*, and potentially also in *P. knowlesi*, which has *sicavar* genes rather than *var* genes [23]. (Analysis of *P. knowlesi*, however, was complicated by two factors – this genome is much less G/C-poor than *P. falciparum*, leading to a higher density of G-rich motifs overall, and it also contains, scattered throughout its chromosomes, many repeats of telomere sequence containing repeated guanine triads that are inherently G4-forming.) Finally, we had observed that in the rodent malaria species *P. berghei*, which has no *var* genes but does encode *pir* genes, G4-forming motifs were co-distributed with the *pir*s, albeit less strongly than was observed with *var*s [23].

This work raised further questions that could not be adequately answered with the genomes available at the time, but can now be addressed due to the recent publication of well-assembled, long-read sequences of the genomes of all seven known Laveranian species [5]. *P. adleri* and *P. gaboni* are relatively ancient ape parasites termed ‘Clade A’ Laverania; *P. billcollinsi, P. blacklocki, P. reichenowi* and *P. praefalciparum* are closer relatives of *P. falciparum* termed ‘Clade B’ (Fig. 1). We investigated whether the G4-*var* association is conserved across Laverania, and the additional possibility that G4s in some Laverania could be co-distributed with other variant gene families as well as (or instead of) *var*s. Notably, although the *var* gene family is uniquely and ubiquitously present in Laverania, its copy numbers vary by 3–4 times across the subgenus, and further clade-specific expansions or contractions of gene families have also occurred: for example, Clade A Laverania have very few *pirs*, but encode a greatly expanded family of *clags* [5]. It therefore seemed possible that G4s were once associated with more ancient multigene families – as per the *pir*s in *P. berghei* – and that they might still be so in earlier Laveranian species, having only recently evolved to be exclusively co-distributed with *var* genes in species such as *P. falciparum*. If true, this would have interesting implications for G4-forming motifs as potential ‘markers’ of variant gene families that experience heavy selective pressure to evolve via regular recombination.

Conducting this analysis raised a second important question: how best to predict G4-forming motifs in *Plasmodium* genomes? Several generations of genome-scanning algorithms now exist for this purpose, but all have been primarily trained – and tested – on G/C/A/T-balanced genomes such as the human genome. It was important therefore to establish how different predictive algorithms perform on extremely G/C-poor genomes, and also to characterize the distribution of ‘canonical’ versus ‘non-canonical’ G4s in these genomes. Canonical motifs take the stereotypical form *G*_3_*N*_*m*_*G*_3_*N*_*m*_*G*_3_*N*_*m*_*G*_3_, while ‘non-canonical’ G4s can, for example, have two rather than three G-quartets, or individual guanines can be missing or interspersed with non-G bases, to give ‘mismatched’ or ‘bulged’ G4s. It is now recognized that non-canonical as well as canonical motifs can fold [25, 26], and that folding depends partly on contextual factors such as *G-richness* (the fraction of Gs in the sequence) and *G-skewness* (the degree of G/C asymmetry between the complementary strands) [27]. Therefore, we compared the performance of two different G4 prediction algorithms, *QGRS Mapper* [28] and *G4 Hunter* [27], in *Plasmodium* genomes. *QGRS Mapper* is a second-generation algorithm that searches for canonical G4-forming sequences, while *G4 Hunter* takes into account G-richness and G-skewness to calculate a score reflecting G4-forming propensity, thus allowing the detection of non-canonical G4s. In addition to comparing the two algorithms, we compared their outputs with recently-published data from G4-seq performed on the *P. falciparum* genome [29]. In contrast to in silico algorithms, G4-seq predicts G4 motifs by sequencing DNA under conditions in which G4s can fold, resulting in characteristic sequencing errors [25]. Overall, a surprising degree of non-concordance appeared between the three approaches, in all the *Plasmodium* genomes that we analyzed.

## Results

### G-quadruplex-forming motifs in all Laveranian genomes are associated with *var* genes

**Fig. 2:**
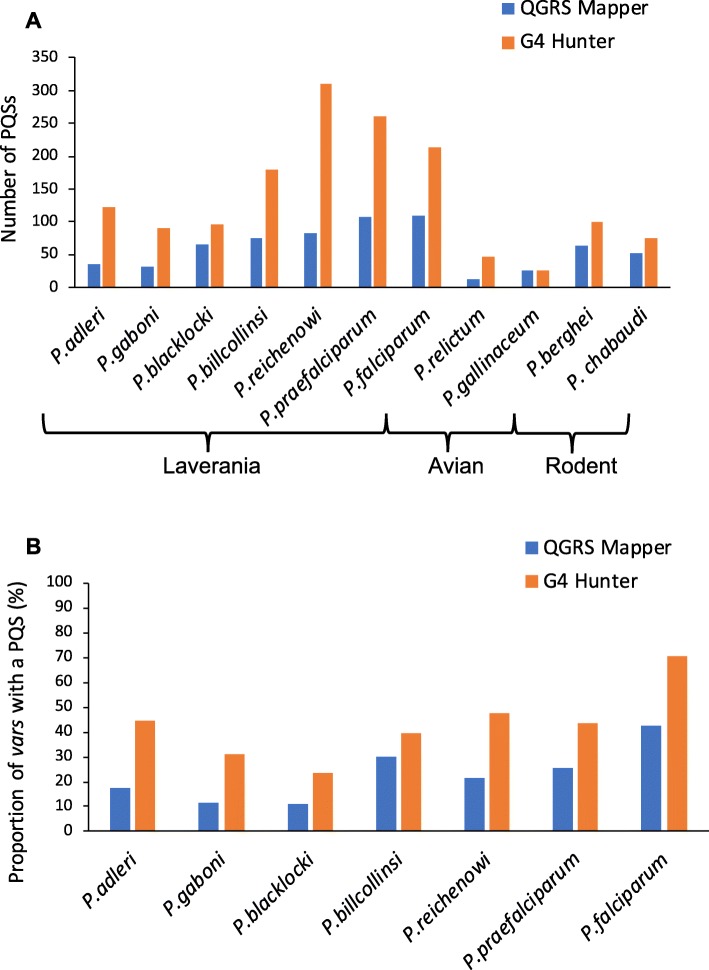
Comparison of total PQSs and *var*-associated PQSs among Laveranian genomes. Bar plots compare the total number of PQSs in Laveranian genomes, as calculated with two different algorithms (**a**), and the number of *var* genes with at least one PQS as a percentage of the total number of *var* genes (**b**). PQSs were considered “*var*-associated” if they were found within a *var* coding sequence, or if the nearest gene, within 2 kb, was a *var* gene. Copy numbers of *var* genes were found in PlasmoDB. Telomeric-PQSs were excluded

In the *P. falciparum* genome, a search for putative quadruplex-forming sequences (PQSs) using *QGRS Mapper* was published in 2016, finding 80 PQSs, of which 35 were *var*-associated [23]. We repeated the search for PQSs in all members of the Laveranian subgenus, including *P. falciparum*, using two PQS prediction tools, *QGRS Mapper* and *G4 Hunter* (Fig. 2a). For *QGRS Mapper*, we used the same parameters as the previous publication (regex: *G*_3_*N*_(0 − 11)_*G*_3_*N*_(0 − 11)_*G*_3_*N*_(0 − 11)_*G*_3_, max length: 45, min G-group: 3, loop size: 0–11). Thus, only ‘canonical’ sequences were identified, with a moderately relaxed loop size of up to 11 nucleotides. For *G4 Hunter*, we used the following parameters: threshold: 1.7, window size: 25, csv data: grouped. This is considered a stringent threshold, at which the ‘precision’ of *G4 Hunter* (i.e. the proportion of genuine G4-forming sequences that are designated as such) should be similar to that of *QGRS Mapper*; the false positive rate is extremely low and the false negative rate is accordingly high (~ 50% of 392 experimentally-confirmed G4-forming sequences were identified at this threshold) [27].

Consistent with the methodology of the 2016 study, we then sought PQSs that were either inside a *var* coding sequence or within 2 kb upstream or downstream of a *var* gene (and with no other nearer gene): these were defined as *var-*gene-associated PQSs. PQSs found in telomere repeats (GGGTT(C/T)A), which comprise the majority of PQSs in any *Plasmodium* genome, were treated separately and never considered *var*-associated.

Despite having similar genome sizes and similar G/C contents (Additional file 1: Table S1), the numbers of PQSs in the Laveranian genomes varied widely: 60 ± 32 with *QGRS Mapper* and 138 ± 91 with *G4 Hunter* (Fig. 2a, Additional file 2: Table S2). Importantly, this was not simply due to differences in how extensively telomere repeats had been assembled in the different genomes, because these repeats were excluded. On average, the Clade B Laverania had more PQSs than the Clade A species (Welch’s t-test, two-tailed, *QGRS Mapper*: t-stat = 6.03, *p* = 0.0025; *G4 Hunter*: t-stat = 2.63, *p* = 0.047). Members of the subgenus differed in their proportions of *var*-associated PQSs (*QGRS Mapper*: 23 ± 11.2%, *G4 Hunter*: 43 ± 14.9%) (Fig. 2b), but these differences did not seem to map onto any particular evolutionary pattern. The differences could not be accounted for by differential G/C content within the *var* families, as this was similar throughout the subgenus (Additional file 1: Table S1). Despite the differences, however, all Laveranian species demonstrated a significant co-distribution of PQSs and *var* genes, compared to the expected distribution in a simulated genome in which *var* genes and PQSs occur at random (Additional file 3: Table S3). Indeed, in *P. falciparum* the *QGRS Mapper* algorithm found PQSs associated with 43% of *var* genes and the *G4 Hunter* algorithm (which tends to find more PQSs than *QGRS Mappe*r) found PQSs in 70% of all *var* genes – even when very stringently applied. This percentage would rise markedly if less stringent thresholds were applied so the great majority of *var* genes might reasonably be predicted to contain putative G4(s).

Overall, this confirms that the co-distribution of PQSs with *var* genes, previously reported only in two closely related Laveranian species separated by ~ 0.19 million years, *P. falciparum* and *P. reichenowi* [23], is actually conserved across all available Laveranian genomes, spanning at least 1 million years of evolutionary time [5]. Furthermore, in *P. falciparum* it was clear that many more PQSs were maintained in real *var* genes than in pseudogenes (Additional file 2: Table S2), adding further weight to the hypothesis that PQSs are positively selected in functional *var* genes.

### Some arrangements of *var*-associated PQSs are conserved in Laveranian genomes, but PQSs in upsB-*var* promoters are unique to *P. falciparum*

**Fig. 3:**
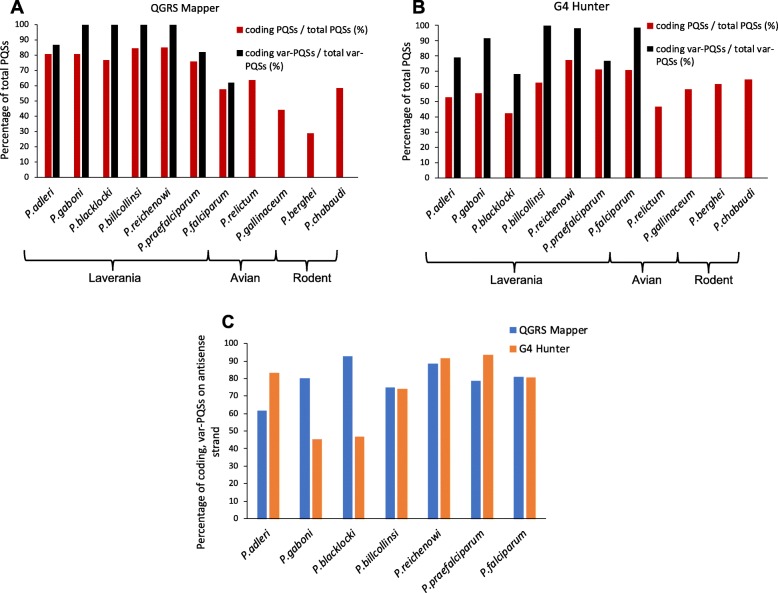
Sense/antisense and coding/noncoding apportionments of PQSs in Laveranian genomes. Bar plots show coding/non-coding and sense/antisense apportionments of PQSs in Laveranian genomes. PQSs were considered ‘antisense’ if they were on the opposite strand relative to their nearest gene. Telomeric-PQSs were excluded. Panels **a** and **b** show the number of coding-PQSs as a percentage of total PQSs (red), and the number of coding, *var*-associated PQSs as a percentage of total *var*-associated PQSs (black), found by *QGRS Mapper* (A) and *G4 Hunter* (B). Panel **c** shows the number of coding, antisense, *var-*associated PQSs as a percentage of total coding, *var*-associated PQSs, found by *QGRS Mapper* (blue) and *G4 Hunter* (orange). For each algorithm, the difference between the percentage of coding, *var-*associated PQSs that were sense versus antisense was assessed (*QGRS Mapper*: t-stat = 11.80, *p*-value = 5.81 × 10^− 8^; *G4 Hunter*: t-stat = 4.43, p-value = 8.19 × 10^− 4^)

We further investigated the relationship between PQSs and *var* genes in the Laverania by searching for conserved arrangements of PQSs in and near *var* genes. First, we noted that in all genomes, the majority of *var*-associated PQSs were within coding regions (Fig. 3a and b, black bars), though this proportion varied from approximately 60% in *P. falciparum* to nearly 100% in other species (*QGRS* Mapper data, Fig. 3a). This bias towards the coding regions of *var* genes was stronger than the genome-wide bias for PQSs to appear in coding regions (Fig. 3a and b, red bars). Additionally, most of the coding *var*-PQSs were on the antisense strand (Fig. 3c). Given that there was no apparent strand bias for PQSs overall – either in the genomes as a whole, or in the non-coding UTRs of *var* genes – this pattern appeared to be specific to the coding location within the *var* gene.

**Fig. 4:**
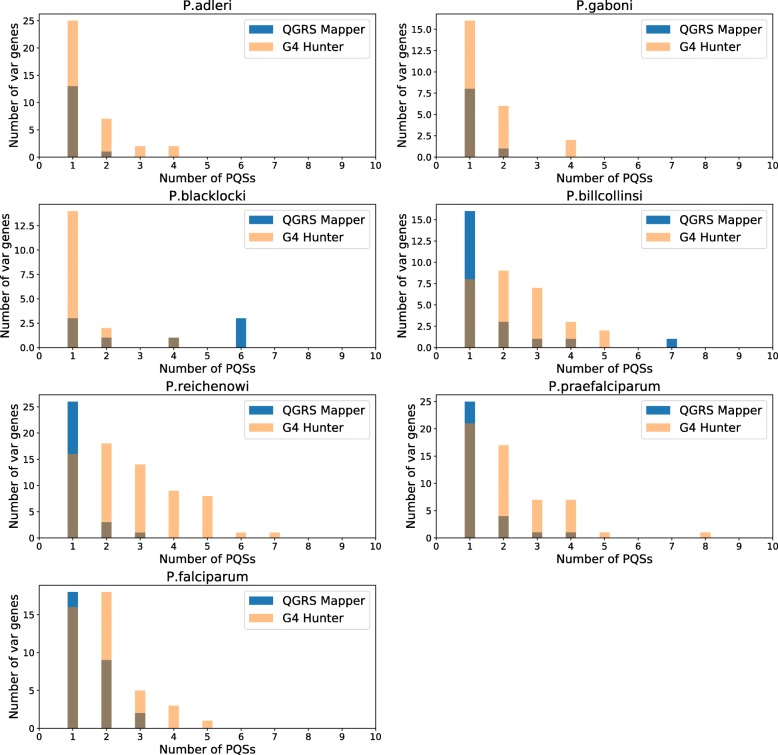
Per-*var* apportionments of PQSs in Laveranian genomes. Histograms show distributions of the number of PQSs per *var* gene in Laveranian genomes, found by *QGRS Mapper* (blue) and *G4 Hunter* (orange). A third colour (brown) is shown where the two distributions overlap. *Var* genes without any associated PQSs are not shown

Next, we analyzed the per-*var* distribution of PQSs, since it was clear that some *var* genes contained more than one PQS (this is statistically unlikely in these extremely PQS-poor genomes, and emphasizes the apparent evolutionary pressure to maintain PQSs associated with *var* genes). We therefore determined whether *var* genes were more likely to have multiple PQSs within them in some species than in others (Fig. 4). Most species tended to have one or two PQSs per *var*, and *G4 Hunter* predicted more PQSs per *var* than *QGRS Mapper*, in line with the general trend (seen in Fig. 2). Furthermore, the data from *G4 Hunter* suggested that the Laveranian species may be evolving towards having more PQSs per *var*, as the majority of *vars* in the Clade A Laverania had one PQS, whereas the Clade B Laverania had a higher proportion of several-PQS *var* genes. *P. blacklocki*, which falls within Clade B but is the most evolutionary ancient in this clade, and thus ‘closest’ to Clade A, has a PQS distribution more closely resembling that seen in the Clade A *var* genes.

**Fig. 5:**
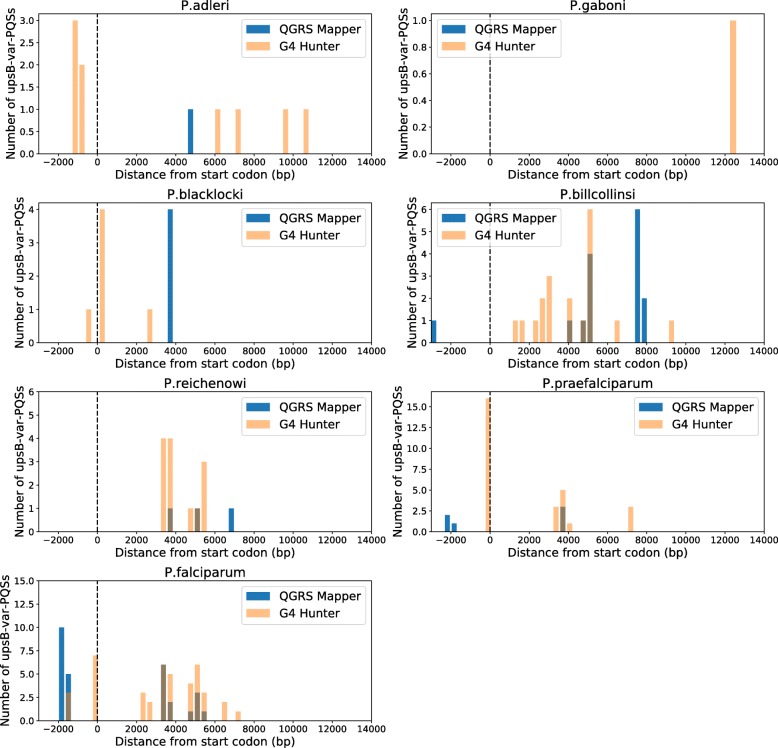
Locations of PQSs within upsB-*var* genes in Laveranian genomes. Histograms show distributions of the locations of upsB-*var*-associated PQSs found by *QGRS Mapper* (blue) and *G4 Hunter* (orange). A third colour (brown) is shown where the two distributions overlap. Locations are reported in terms of distance from the start codon (denoted by the vertical dashed line at a distance of “0”)

In their previous search for PQSs in *P. falciparum* and *P. reichenowi*, Stanton et al. reported that almost half of the *var*-associated PQSs in *P. falciparum* were located upstream of upsB-type *var* genes (an observation first made by Smargiassio et al. [17]), whereas this group was entirely absent in *P. reichenowi*, and almost all of the *var*-associated PQSs in *P. reichenowi* were coding [23]. ‘UpsB’ refers to a subgroup of the *var* gene family, which is frequently sub-classified according to similarity in the upstream (‘ups’) regions of the genes: upsB-type *var* genes are the largest subgroup in *P. falciparum* and are found immediately inside the telomeres of almost every chromosome, placing their upstream region directly adjacent to the subtelomeric repeats. We repeated this analysis for the newly published Laveranian genomes to determine whether the clustering of PQSs in upsB-*var* promoters was unique to *P. falciparum*. In our analysis, most *var*-PQSs in *P. falciparum* were indeed associated with upsB-type genes (62 and 44% of the total *var-*PQSs with *QGRS Mapper* and *G4 Hunter*, respectively), in concordance with the findings of Stanton et al. [23]. Other Laveranian species, including *P. reichenowi*, did have some upsB-*var*-PQSs, but they constituted less than 20% of the total (Additional file 4: Fig. S1), and were usually in coding regions rather than 5′ UTRs (Fig. 5). Finally, we noticed a prominent cluster of PQSs in most Clade-B species that was located ~ 5 kb downstream of upsB-*var* start codons (Fig. 5). Thus, there is evidently a conserved bias in the location of PQSs within the coding sequences of these genes, but the non-coding, upstream PQSs are not conserved and appear to be unique to *P. falciparum*.

### G-quadruplex-forming motifs in Laveranian genomes are not strongly associated with other multigene families

Stanton et al. previously reported an association between PQSs and *pir* genes in *P. berghei* [23], suggesting that PQSs could be co-distributed with variantly expressed virulence gene families in addition to *var*. The *var* gene family is uniquely present among Laverania, but other more ancient multigene families including *pir*, which may also have roles in virulence, have not been lost in these species: they still exist in varying numbers alongside the *var* genes. These other gene families are also highly variable in number: for example, some early Laverania have very few *pirs*, whereas *P. falciparum* has several hundred, but the early species instead have 30+ *clags,* whereas *P. falciparum* has only five [5]. We sought to establish whether any of these other families might be associated with G4s, and whether any evolutionary pattern could thus be discerned.

We searched the Laveranian genomes for co-distributions of PQSs with other multigene families, including *clag, PHIST, pir, rifin,* and *stevor.* Surprisingly, we found very few PQSs in these gene families (Additional file 5: Fig. S2 and Additional file 6: Table S4). Neither algorithm predicted any PQSs in *stevor* genes. The *clag* family showed a weak association with PQSs in *P. blacklocki*, which has a large *clag* family (2 out of 35 *clag*s, i.e., 6%, had PQSs), and also in *P. billcollinsi* and *P. reichenowi*, which have smaller *clag* families (1 PQS amongst 7 and 6 *clag*s respectively), while the *PHIST* family was moderately associated with PQSs in *P. gaboni*, *P. adleri*, *P. billcollinsi*, and *P. praefalciparum* (PQSs were found in 1 or 2 genes, representing 12.5, 22, 20, and 8% of the total *PHIST* genes in each species, respectively). Additionally, some species had PQSs in a few *pir* genes and/or *rifin* genes, but the proportions were always small compared to the total sizes of the gene families. In all cases, the PQSs found in non-*var* multigene families were almost all found by *G4 Hunter* but not by *QGRS Mapper*, indicating that they are probably of non-canonical types. This is in contrast to the *var*-associated PQSs, many of which are canonical (Fig. 2).

### Numbers and distributions of G-quadruplex-forming motifs vary significantly in non-Laveranian Plasmodium genomes

In addition to studying the Laveranian subgenus of *Plasmodium*, we analyzed the genomes of two more distantly related outgroups: species infecting rodents (*P. berghei, P. chabaudi*) and birds (*P. relictum, P. gallinaceum)*. These species do not have *var*s, but do have varying numbers of the other multigene families. We found significantly fewer PQSs in *P. relictum* and *P. gallinaceum* as compared to the Laverania (Welch’s t-test, two-tailed, *QGRS Mapper*: t-stat = 3.92, *p*-value = 0.00718; *G4 Hunter*: t-stat = 4.35, p-value = 0.00355), while *P. berghei* and *P. chabaudi* had approximately the same number of PQSs as the Clade A Laverania (Fig. 2a). *P. gallinaceum* was unique in not having more PQSs found by *G4 Hunter* than *QGRS Mapper*.

**Fig. 6:**
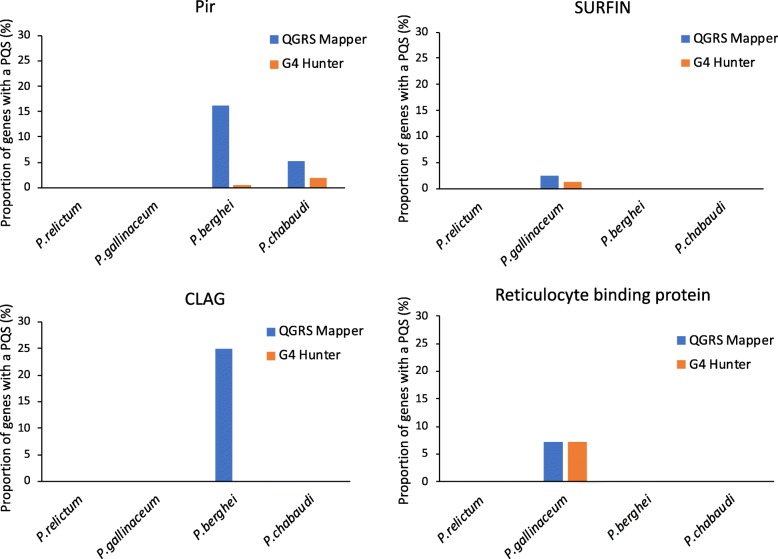
Associations between PQSs and variantly-expressed multigene families in rodent and avian malaria parasites. Bar plots compare the proportion of genes in variantly-expressed, multigene families that contain at least one PQS, among rodent and avian malaria parasites. Data for *QGRS Mapper* are shown in blue; data for *G4 Hunter* are shown in orange. Copy numbers of genes in each family were found in PlasmoDB

By searching these genomes for associations between PQSs and multigene families (*clag, PHIST, pir, rifin, stevor*, reticulocyte-binding (Fig. 6)), we confirmed the co-distribution of PQSs with *pirs* in *P. berghei* that was reported by Stanton et al. [23]: 37 of 217 *pir* genes had PQS(s) according to *QGRS Mapper*, i.e. ~ 17% of all *pirs*, and this constituted over 50% of the total genomic, non-telomeric PQSs. A significant, but markedly weaker, co-distribution was also seen in *P. chabaudi*, with 11 of 208 *pir* genes having PQS(s) (Additional file 3: Table S3). Thus, this association may be common to *pir* genes of rodent malarias, but it is clearly weaker that that seen in *var* genes, and unlike the *var* genes it does not segregate with ‘real’ *pir* genes versus *pir* pseudogenes (pseudogenes are common in the *P. berghei* genome and many PQSs are found in these pseudogenes). Nevertheless, there is a contrast to the Laveranian genomes, where PQSs are rarely, if ever, in *pir*s: the two co-associate spatially simply due to the proximity between *pir* and *var* genes. Notably, in both *P. berghei* and *P. chabaudi*, *pir* genes were exclusively associated with canonical PQSs as found by *QGRS Mapper*: the types of PQSs found by *G4Hunter* showed no association with *pir* genes. No other gene families harboured large numbers of PQSs in *P. berghei*, *P. chabaudi, P. relictum*, or *P. gallinaceum* (in *P. berghei*, one *clag* gene out of 4 harboured a PQS (Fig. 6), but given the small size of the gene family, this may be of limited relevance).

### Some non-var-G4s are conserved in Laveranian genomes

**Fig. 7:**
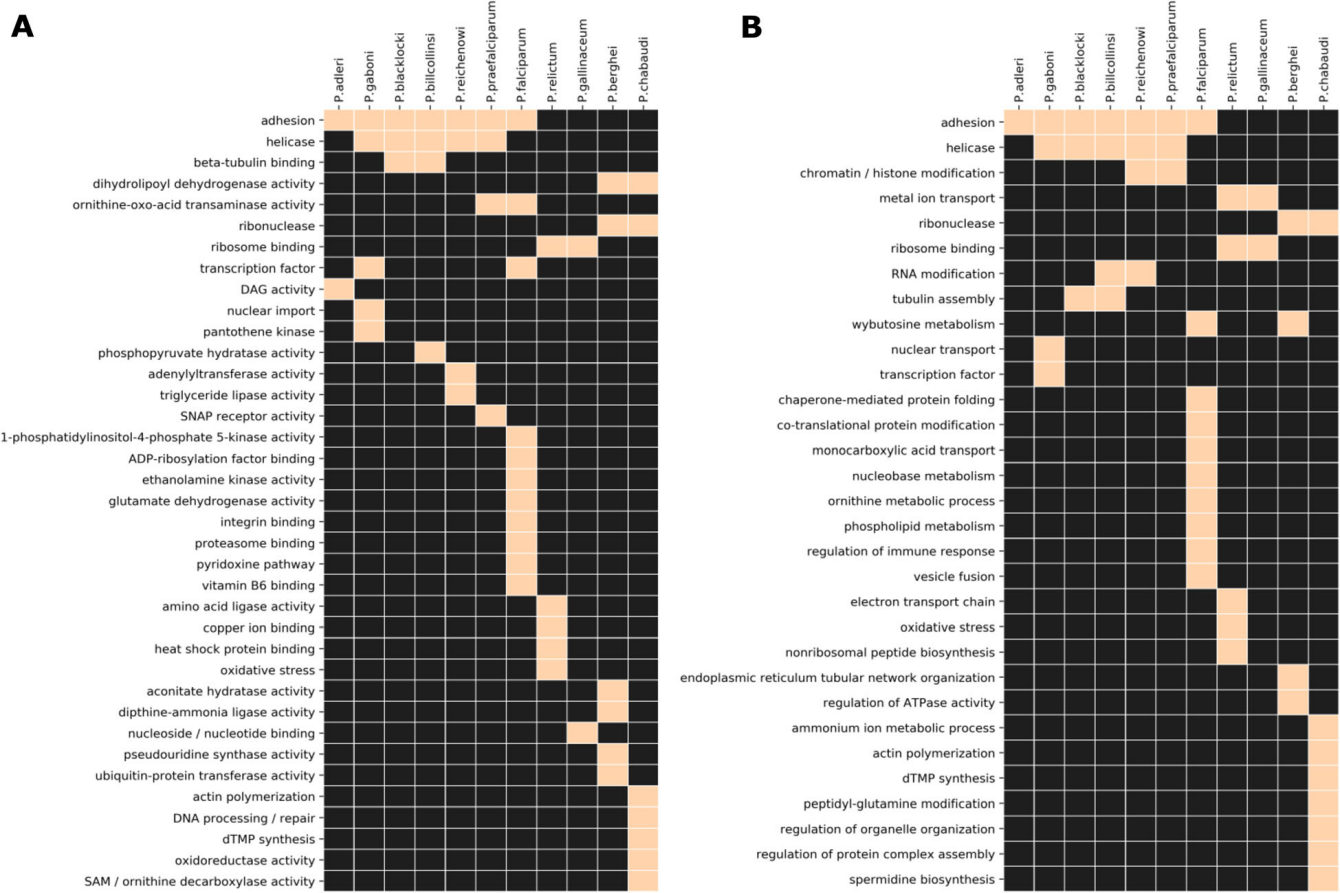
GO terms analysis of PQS-associated genes for Laverania, rodent, and avian malaria parasites. Lists of PQS-associated genes for each species were analyzed for gene ontology (GO) terms in PlasmoDB. The gene lists included PQS-associated genes from both *QGRS Mapper* and *G4 Hunter*. Heat maps show overrepresented GO terms for molecular function (**a**) and biological processes (**b**) for each species. Before constructing the heat maps, similar GO terms were binned together. Any group of GO-terms having PQS-associated genes in a particular species is marked in pink whereas negatives are marked in black. The list of raw GO terms and their binning assignments can be found in Additional file 8: Table S5

Since the Laveranian genomes did not show strong associations between PQSs and multigene families other than *var*, we analyzed the degree to which non-*var* PQSs found anywhere else in these genomes might be conserved. We found that several PQS-gene associations were conserved (Additional file 7: Fig. S3): for example, all Laverania possessed at least one PQS in the genes encoding circumsporozoite protein, alpha/beta hydrolase, AP2 domain transcription factor(s), and SNARE proteins, whereas these associations were absent in the avian- and rodent-infecting *Plasmodium*s. Additionally, all Laverania except *P. reichenowi* possessed at least one PQS in the 40S ribosomal protein S2-encoding gene, and all Laverania except *P. blacklocki* had at least one PQS in a *PHIST* gene. PQS-encoding genes were then clustered by GO-terms to seek any functional groups that might be particularly enriched in PQSs. Fig. 7 shows that the most prominent PQS-encoding genes were clearly the adhesin-encoding genes – i.e. primarily *var*s. A second group of helicase-encoding genes also contained conserved PQSs in all Laverania except *P. falciparum* (Fig. 7 and Additional file 8: Table S5).

### QGRS mapper, G4 hunter and G4-seq all give markedly different results on AT-rich *Plasmodium* genomes

**Fig. 8:**
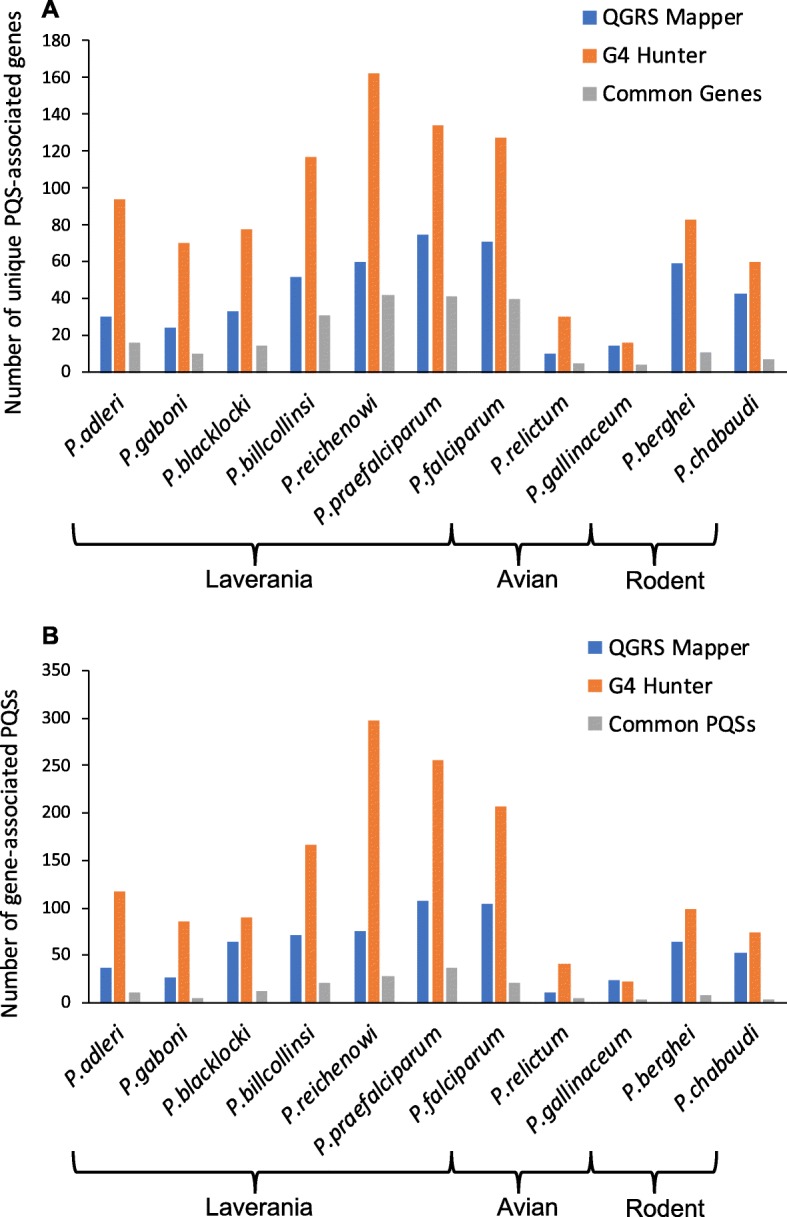
Comparison of *QGRS Mapper* and *G4 Hunter* in their performance on AT-rich *Plasmodium* genomes. Bar plots compare the number of unique PQS-associated genes (**a**) and the number of gene-associated PQSs (**b**) found by *QGRS Mapper* (blue) and *G4 Hunter* (orange) for each *Plasmodium* species. Instances where both algorithms predicted at least one PQS in the same gene were considered “common genes” (A, grey). Instances where both algorithms predicted the same PQS were considered “common PQSs” (B, grey)

Finally, having conducted all the foregoing analyses with two different PQS-prediction algorithms, we compared their concordance. *QGRS Mapper* searches for sequences that match the general motif favorable for G4 formation, *G*_3_*N*_(0 − 11)_*G*_3_*N*_(0 − 11)_*G*_3_*N*_(0 − 11)_*G*_3_, whereas *G4 Hunter* does not require this ‘canonical’ form but considers additional factors such as G-richness and G-skewness to calculate a score that reflects the propensity of a sequence to form a G4. *G4 Hunter* can therefore detect the “non-canonical” types of G4s that do not match the stereotypical pattern and would be overlooked by *QGRS Mapper*. By using both algorithms, we sought to characterize the proportions of canonical and non-canonical PQSs in *Plasmodium* genomes, and to compare the performance of the algorithms in general on AT-rich genomes. For all species except *P. gallinaceum*, *G4 Hunter* found more PQSs than *QGRS Mapper*, though this difference varied among species and did not track with any evolutionary pattern (Fig. 2a). Next, we investigated the degree of concordance between the two algorithms: for each species, we counted the number of PQS motifs, and also the number of unique PQS-containing genes, that were detected by both algorithms. We found a surprising lack of concordance (Fig. 8): only a minority of motifs were found in common. The percentage of common genes was somewhat greater, indicating that although both algorithms might find a PQS in the same gene, they usually did not find exactly the same motif. Concordance also varied considerably between genomes, being relatively high in several Clade B Laverania and particularly low in the rodent and avian malaria genomes.

To test whether the lack of concordance was caused by setting an excessively high threshold for *G4 Hunter*, we re-analysed the *P. falciparum* genome at a lower threshold of 1.25 (this was initially set at 1.7 to reduce the risk of false positives and obtain a highly confident set of PQSs). Using a threshold of 1.25 increased the number of PQSs more than 10-fold, particularly in telomere repeats (Additional file 13: Table 8, and Additional file 9: Fig. S4) but it did not substantially increase concordance. Thus, even at a moderate threshold, *G4 Hunter* does not detect all the canonical motifs detected by *QGRS Mapper*; instead, the two algorithms appear to detect fundamentally different types of PQS. By experimentally testing the G4-folding capacity in three oligonucleotide sequences of new *G4 Hunter* PQSs, alongside two previously-published motifs found by *QGRS Mapper*, we showed that both types of PQS can indeed form G4s, at least in vitro (Additional file 10: Fig. S5)*.*

**Fig. 9:**
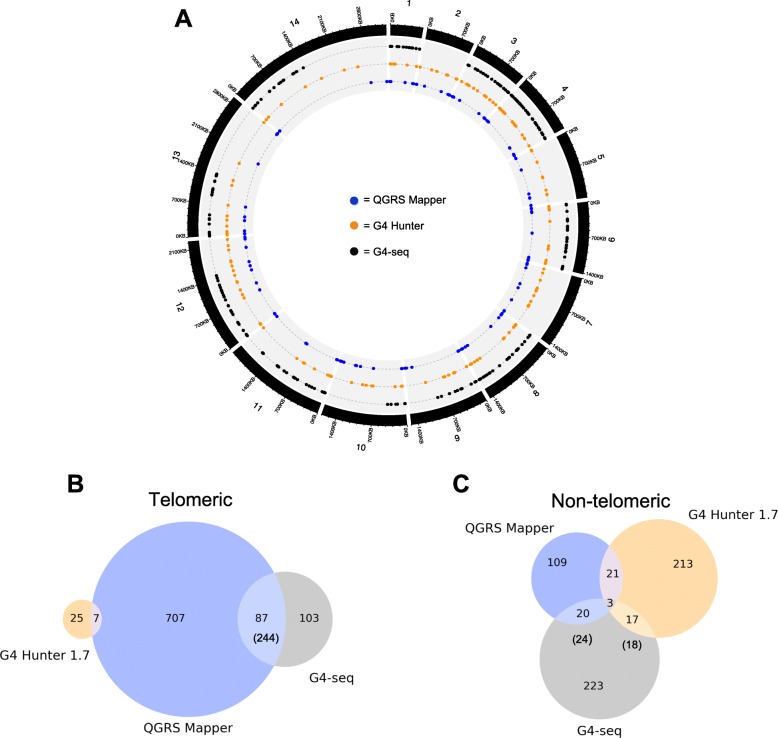
Analysis of concordance among motifs predicted by *QGRS Mapper*, *G4 Hunter* and experimental G4-seq in the *P. falciparum* 3D7 genome. A circos plot (**a**) shows the distribution of PQSs across the 14 chromosomes in the *P. falciparum* 3D7 genome. Data from *QGRS Mapper*, *G4 Hunter* and experimental G4-seq are depicted by blue, orange and black circles, respectively. G4-seq yielded no data for chromosomes 2, 5 and 7 because these chromosomes were not covered in the original sequencing data (GEO accession GSM3003550). Note that telomeric PQSs with similar locations appear to cluster into a single data point, which visually minimizes the number of these PQSs (particularly in the case of *QGRS Mapper*, where many PQSs were detected within telomere repeats). Venn diagrams (**b** and **c**) depict the overlaps between PQS sequences detected by *QGRS Mapper*, *G4 Hunter* (threshold = 1.7), and experimental G4-seq. For G4-seq data, PQSs within 20 kb of chromosomal ends were considered telomeric. Due to the size of the G4-seq windows, it was possible for multiple PQSs to fall within the same window. Numbers in parentheses show the “full” count of PQSs detected within G4-seq windows, while numbers not in parentheses show the number of unique G4-seq windows that contained at least one PQS

We then compared the PQSs found by each algorithm with the locations of G4s that were experimentally observed in G4-seq on the *P. falciparum* genome – this being the only *Plasmodium* genome for which G4-seq has been performed [29] (Fig. 9a). G4-seq identified a total of 173 sites when the genome was sequenced in potassium-containing conditions, and twice as many, 326, in potassium-plus-pyridostatin conditions (pyridostatin is a small molecule that stabilizes G4s and can thus increase the number of G4s that are detectable by sequencing [25]). It is clear that the overall number of sites detectable by G4-seq was greater than the number of unique (non-telomere) PQSs predicted by either in silico algorithm, but in fact > 80% of all sites found by G4-seq in the *P. falciparum* genome proved to be in telomeres, so only a small fraction of the predicted non-telomeric PQSs were actually found by sequencing. Fewer than one third of the total G4-seq sites had the potential to be predicted by *QGRS Mapper* using a loop size of 0–11, because only 32.5% of the sequenced sites were canonical (defined by Marsico et al. as containing a *G*_3_*N*_(0 − 12)_*G*_3_*N*_(0 − 12)_*G*_3_*N*_(0 − 12)_*G*_3_, motif) [29]. In fact, *QGRS Mapper* predicted 24 unique, non-telomere-repeat sites that appeared in G4-seq (it did also predict a further 244 sites containing telomere repeats that were also picked up by G4-seq) (Fig. 9b,c). Excluding telomere repeats, however, only 22% of the total *QGRS Mapper* predictions for canonical G4s were verified by G4-seq. *G4 Hunter*, by contrast, could potentially have predicted any of the sequenced sites, but in fact predicted only 18 of them, so just 8.1% of *G4 Hunter* predictions were verified by G4-seq (Fig. 9c, Additional file 11: Table S6).

## Discussion

Our study shows that across all known Laveranian species, the co-distribution of G-quadruplex-forming motifs with *var* genes is strongly conserved. This was previously reported only in two closely-related species, *P. falciparum* and *P. reichenowi*, which have very similar numbers and arrangements of *var* genes [23]; we now show that it remains true across all seven species and at least five times as much evolutionary time, despite striking interspecies differences in the total numbers of *var* genes, the total numbers of PQSs and indeed the proportions of *var*-associated PQSs. Notably, Clade B Laverania had more PQSs overall in their genomes than Clade A species, suggesting that selection for these motifs may have increased rather than decreased across the evolutionary pathway. This did not appear to be exclusively due to strengthening selection for PQSs in *var* genes, because *var*s in Clade B were not universally more associated with PQSs than those in Clade A, nor did the species with the largest *var* gene families have the most numerous PQSs. However, we were unable to account for the differences by associating PQSs with any other variant gene family, or with any other gene or gene-class elsewhere in the genome. Overall, therefore, it appears that PQSs have been uniquely selected to associate with *var* genes across the Laverania. There are many possible reasons for this, but at present the main reason supported by experimental data is that G4s can promote beneficial diversity-generating recombination between *var* genes [23, 30].

We next examined in detail the distribution and location of PQSs in and around *var* genes. We observed that several *var* genes had more than one PQS – an unlikely event if these sparse motifs were randomly distributed across the genome, and one that emphasizes the apparent selection for PQSs in *var* genes. Furthermore, Clade B Laveranian species may be evolving towards having more PQSs per *var*, since PQS clusters were more common in *var* genes in Clade B than Clade A, and *P. blacklocki*, the most evolutionarily ancient species in Clade B, had a PQS distribution most closely resembling that seen in the Clade A *var*s. Secondly, the great majority of PQSs were in coding rather than UTR regions, with a distinct bias for the antisense strand. This could potentially allow the *var* genes to benefit from the recombination-promoting effects of G4s without suffering any interruption of coding-strand transcription, which could impede efficient production of PfEMP1. (We have previously showed that a stabilized coding-strand G4 can inhibit transcription of a *rifin* reporter gene [31], so the same could be true for G4-encoding *var* genes.) Coding PQSs – at least amongst the upsB *var* genes that were analysed in Fig. 5 – occurred mostly around the middle of *var* gene sequences, within the second exon that encodes the variable extracellular domain of PfEMP1. This would maximize the potential for DNA breaks resulting in beneficial domain-shuffling between *var* genes, which is known to occur in an organized and conservative manner in *P. falciparum* [15]. The conserved organization across the Clade B Laverania would again suggest that PQSs may function similarly in all these genomes. Thirdly, *P. falciparum* encoded an additional large set of PQSs upstream of upsB-type *var* genes, which appeared to be unique – i.e. not observed in other Laverania. It is intriguing to speculate that this may be a recent evolution in this species, perhaps selected to play a new role in transcriptional control of *var* genes (a known function of G4s upstream of oncogenes [19]). Alternatively, these G4s could buffer the transmission of chromatin states along DNA between the telomeres and the upsB genes [32].

Moving from the *var* genes to other variant gene families, we failed to find any strong association of PQSs with any other particular gene family in the Laverania. It was not the case, for example, that the strikingly expanded *clag* families in the more ancient species all harboured high numbers of PQSs. We did observe a few *clag* and *PHIST* genes that contained a (non-canonical) PQS but any biological significance of this remains unclear.

In two outgroups – avian and rodent malarias – we sought associations between PQSs and variant gene families that might hint at how the situation in the Laverania evolved, and furthermore might support the idea that G4s could play the same role in promoting recombination and diversification in any gene family that is under immune pressure. In *P. berghei* and *P. chabaudi*, PQSs were co-distributed with *pir* genes – albeit relatively weakly and only for canonical (*QGRS Mapper*) PQSs. There is indeed some evidence that *pir* genes encode variantly expressed virulence determinants that could theoretically benefit from mitotic diversification, analogous to that of the *var* genes [8, 9]. Alternatively, it is possible that the G4s might act as transcriptional modulators, since the majority of *pir*-associated PQSs in *P. berghei* were non-coding, in contrast to the coding exon-2 bias of PQSs within *var* genes.

PQSs in the avian-malaria genomes were notably sparse, over and above what might be expected from their underlying genome bias. At 17.0 and 18.3% G/C, *P. gallinaceum* and *P. relictum* are both lower than *P. falciparum* (19.3%), but comparable to several other Laveranian genomes (17.6–18.6%) which still harbour twice as many PQSs as either avian genome. The few PQSs that *were* found were not strongly associated with any gene family (*clag, PHIST, pir, rifin* or *stevor*) so, if the above theory is correct, it may be that no gene family in these avian parasites has evolved to diversify via G4-mediated recombination.

Besides the variant gene families, about half of the total PQSs in most Laveranian genomes were associated with other genes throughout the genome, so we assessed the conservation of these motifs as well. This proved to be limited: although certain genes such as the gene encoding circumsporozoite protein were clearly conserved complete with their PQS across the Laverania, only two functional groups of genes showed a highly conserved enrichment of PQSs and one of these was the *var*s (the other being helicase-encoding genes, see Fig. 7).

Finally, we compared *QGRS Mapper* and *G4 Hunter* in their performance on G/C-poor genomes, and found a surprising lack of concordance between the two algorithms. It was expected that *G4 Hunter* would find greater numbers of PQSs, due to its ability to accommodate non-canonical sequences, and the same has indeed been reported in other genomes [27]. However, *G4 Hunter* did not simply find all the canonical sequences and then some additional non-canonical ones. In some genomes, such as *P. relictum, G4 Hunter* did find 50% of the canonical sequences, but in *P. chabaudi* it found only ~ 8%. In fact, *P. gallinaceum*, which had extremely few PQSs overall, was unique in having the same numbers of PQSs found by both algorithms, but again, they were generally not the same motifs. This suggests that in some genomes, many or most of the canonical PQSs are not actually predicted to fold under stringent *G4 Hunter* conditions, perhaps due to long loop sizes or unfavourable G-richness or G-skewness in their sequence context. This is not unprecedented: the originators of *G4 Hunter* reported that canonical PQSs in the *myc* promoter were not necessarily predicted by stringently-thresholded *G4 Hunter* [27]. Amongst *Plasmodium*, this phenomenon was much more marked in the outgroups than in the Laverania, perhaps suggesting that canonical G4s that actually do fold are positively selected in Laveranian genomes – consistent with the proposal that stable, folded G4s play a particular beneficial role in *var* gene organization.

Our analysis emphasizes that it is clearly important to check any in silico prediction experimentally. Very few *Plasmodium* sequences have been thus verified, and the two that have were selected a priori by *QGRS Mapper*. (Notably, neither of them – belonging to the *rifin* gene *PF3D7_0700200* and the *var* upsB region [17, 31] – were found here by *G4 Hunter*: they presumably fall into the false negative category which is expected to be quite large at a threshold of 1.7.) Here, we experimentally tested three new *G4 Hunter* motifs, including two with the apparent potential to form only two-quartet rather than three-quartet G4s. All three were found to bind to the G4-specific dye thioflavin T, suggesting that PQSs found by *G4 Hunter* can fold as readily as those found by *QGRS Mapper*. The two algorithms appear to find fundamentally different types of motif, and should perhaps be viewed as complementary tools for G4 prediction.

Besides the biophysical characterization of individual sequences, genome-wide G4-seq has recently been conducted in *P. falciparum* [29] and this is an experimental rather than in silico method, albeit still performed in vitro on isolated DNA. In the human genome, G4-seq found more G4s than the original number predicted in silico, and many of those were non-canonical [25]. Similarly, more than two-thirds of the G4-seq sites found in the *P. falciparum* genome were ‘non-canonical’ [29] – an even greater proportion than in the human genome – but they were predominantly not the sites predicted by *G4 Hunter*, with less than 10% overlap between the *G4 Hunter* predictions and the G4-seq sites. This raises several important points. Firstly, *Plasmodium* genomes probably harbour a high proportion of non-canonical G4s compared to the human genome, with potential implications for a) their stable folding in DNA versus RNA (2-quartet motifs are expected to be more stable in RNA), and b) for the proteins that might metabolize these structures. Secondly, the ‘state of the art’ algorithm *G4 Hunter*, at least when stringently applied, may not perform very accurately on the unusual PQS content of very G/C-poor genomes. Unexpectedly, the second-generation algorithm *QGRS Mapper* actually did better: 20% of its predicted sites were found to be folded in G4-seq. Nevertheless, this is still a low proportion, and *QGRS Mapper* can predict only a minority of G4-seq sites in any genome where they are mostly non-canonical. Thirdly, caution is therefore advisable when using any in silico algorithm to predict G4s in *Plasmodium* genomes and experimental validation is especially important – in vitro and ideally *in cellulo.*

## Conclusion

This research shows that G4-forming motifs are uniquely strongly associated with *Plasmodium var* genes, suggesting that Laveranian malaria parasites have evolved a particular role for G4s in the organization of *var* genes. Secondly, in the A/T-rich genomes of *Plasmodium* species, the choice of prediction algorithm may be particularly influential when studying G4s in these important protozoan pathogens.

## Methods

### Organisms and genomic information

Genome sequences of the genus *Plasmodium* were downloaded from PlasmoDB release 44 [33]. The following *Plasmodium* strains were used for analysis: *P. adleri* G01, *P. gaboni* G01, *P. billcollinsi* G01, *P. blacklocki* G01, *P. reichenowi* G01, *P. praefalciparum* G01, *P. falciparum* 3D7, *P. relictum* SGS1-like, *P. gallinaceum* 8A, *P. berghei* ANKA, and *P. chabaudi* chabaudi.

### In silico prediction of PQSs in *Plasmodium* genomes using QGRS Mapper and G4 Hunter

PQSs were found throughout this work using the tools *QGRS Mapper* (version 1) [28] and *G4 Hunter* [27]. The parameters used for the two algorithms were: *QGRS Mapper*- regex: *G*_3_*N*_(0 − 11)_*G*_3_*N*_(0 − 11)_*G*_3_*N*_(0 − 11)_*G*_3_, max length: 45, min G-group: 3, loop size: 0–11; *G4 Hunter*- threshold: 1.7, window size: 25, csv data: grouped. Bedrat et al. [27] used similar parameters while comparing the performance of the *quadparser* and *G4 Hunter* algorithms on the human mitochondrial genome: they found that these yielded low false positive rates while also predicting comparable numbers of PQSs. An additional analysis of the *P. falciparum* genome was conducted with a *G4 Hunter*-threshold of 1.25 instead of 1.7.

For each species, each chromosome sequence was downloaded from PlasmoDB and analyzed using the *QGRS Mapper* and *G4 Hunter* web applications. *QGRS Mapper* does not simultaneously analyze both the sense and antisense strands, so reverse-complements of the chromosomes and contigs were generated and analyzed separately by this algorithm. For genomes with large numbers of contigs (> 5), the contig sequences were stripped of their FASTA headers, binned into a single .txt file, and analyzed together. Each PQS was then assigned to its associated contig by searching for the sequence in the list of contig sequences. Full lists of the sequence motifs found with each algorithm are tabulated in Additional files 12 and 13 (Tables S7 and S8).

### Parsing genomic coding sequences for PQS-associated gene annotations

Files containing annotated coding sequences in FASTA format for each species were downloaded from PlasmoDB release 44. For each PQS location, the nearest gene ID and its annotation were found by parsing the list of coding sequences for the species. The distance between each PQS and the nearest gene was measured from the start of the PQS to the start or end of the coding sequence. If the PQS was found within a coding sequence, a distance of “0” was assigned. If the PQS location was downstream of the nearest gene, the distance was represented as a negative integer.

### Classification of telomeric and gene-associated PQSs

PQSs predicted by *QGRS Mapper* and *G4 Hunter* were further classified as telomeric, gene-associated, and/or upsB-*var* gene-associated. Telomeric PQSs were identified as those with repeating 7-bp motifs (GGGTTT/CA), which are characteristic of telomeric sequences in *Plasmodium spp*. [34]. These telomeric PQSs were excluded from downstream analyses. The remaining, non-telomeric PQSs were classified as “gene-associated” if they were found within 2 kb of the beginning or end of a genomic coding sequence. An additional classification was made for PQSs that were associated with upsB-type *var* genes. Since the gene annotations in the v.44 assemblies did not specify particular *var* classes (e.g. ups-A, ups-B, ups-C), the assignments were made on the basis of gene location and orientation. upsB-type *var* genes were defined as those at the far ends of the chromosomes and transcribed inwards (with the 5′ UTR nearest the telomere).

### Statistical analysis of the co-distribution of PQSs and multigene families

Statistically significant associations between the locations of *var*/*pir* genes and PQSs were determined as per Stanton *et. al.* 2016 [23]. Briefly, the distance of every gene from its nearest PQS was calculated, measuring from the start or end of the PQS and scoring ‘0’ for genes with a PQS within their coding sequence. A set of genes with random positions was then simulated as a comparator to test the null hypothesis of no association. The actual dataset and the simulated datasets were analyzed to determine the mean and median distance from PQSs, with the actual distance being compared to the null situation. The statistical significance of each mean difference was assessed using Welch’s t-test (2-tailed), with significance set at *p* ≤ 0.05, or for each median distance, by calculating a 95% confidence interval around the median.

For genome assemblies that contained contigs, these were treated as discrete chromosomes. Additionally, *vars/pirs* on chromosomes and contigs that did not contain any PQSs had to be excluded, since the ‘distance to nearest PQS’ did not exist. In *P. berghei* and *P. chabaudi*, which each encode an isolated group of *pir* genes more than 1 Mb from the nearest PQS, these marked outliers were excluded.

### Gene ontology (GO) term enrichment analysis of PQS-associated genes

For each species, a list of PQS-associated genes was compiled from the *QGRS Mapper* and *G4 Hunter* datasets. The lists were analyzed for enriched GO terms using the Gene Ontology Enrichment tool in PlasmoDB [33], with parameters: ontology: molecular function and biological process, evidence: computed and curated, limit to GO Slim terms: No, *p*-value cutoff: 0.05. Similar GO terms from the resulting datasets were binned together (Additional file 8: Table S5) and visualized with heatmaps, which were constructed using the Seaborn library in Jupyter Notebook.

### Comparing QGRS Mapper, G4 Hunter, and G4-seq in their analysis of AT-rich *Plasmodium* genomes

*QGRS Mapper* and *G4 Hunter* were compared by searching for instances where both algorithms predicted the same PQS, or where the PQS of one algorithm was a “substring” of a sequence predicted by the other algorithm. The in silico predictions were compared to experimental G4-seq data [29] by searching for instances where PQS start/end locations overlapped with G4-seq window locations. A circos plot of PQS locations was constructed using the shinyCircos library in R Studio, and Venn diagrams were constructed using the venn3 library in Python 3 [35].

### Statistical testing for comparison of means and distributions

Comparisons between Clade A Laverania, Clade B Laverania, rodent malaria parasites and avian malaria parasites often involved groups with unequal sample sizes and variances. In these cases, the two-sided Welch’s t-test was chosen, since it makes no assumptions regarding equality of sample sizes and variances. Descriptive statistics were calculated using Microsoft Excel. Hypothesis testing was performed using the Scipy library in Python 3. All statistical tests were interpreted at a significance level of 0.05.

### G4 folding assay

PQS oligonucleotides were tested for their ability to fold into G4s using the G4-specific fluorescent dye thioflavin T [36]. Each oligonucleotide was tested alongside a sequence-scrambled version. 20 μM oligonucleotides were heated to 90 °C for 5 mins and buffer was added at 100 μM Tris pH 7.8, 100 μM KCl. Cooling from 90 °C to room temperature was then controlled at a rate of 5 °C/5 mins. 80 μM thioflavin T (Sigma) was added and incubated at room temperature for 5 mins. 40 μl of each sample was transferred in triplicate to a 96-well, black, Uclear plate (Greiner) and fluorescence was measured (Ex. 420 nm, Em. 480 nm) using a FLUOstar Omega plate reader (BMG Labtech).

### Custom code for data collection and analysis

All parsing was performed using Python 3.6.5. Additional data analysis, visualization, and hypothesis testing were performed using Jupyter Notebook. Code is available at https://github.com/hgage/G4s-across-Plasmodium-evolution.

## Supplementary information

**Additional file 1 Table S1.** Genome sizes and G/C content of *Plasmodium* parasites. Genome sequences were downloaded from PlasmoDB v. 44. Genome sizes and G/C contents were calculated using custom code. Custom code was used to parse the coding sequences for *var* genes and to calculate their associated G/C contents.

**Additional file 2 Table S2.** Comparison of total PQSs and *var*-associated PQSs in Laveranian genomes. PQSs were considered “*var*-associated” if they were found within a *var* coding sequence, or if the nearest gene, within 2 kbp, was a *var* gene. Copy numbers of *var* genes were found in PlasmoDB. Telomeric-PQSs were excluded.

**Additional file 3 Table S3.** Co-distribution of PQSs with variantly-expressed multigene families in Laverania, rodent, and avian malaria parasites. For each genome, the mean distance between *var* or *pir* genes and their nearest PQS was compared to the mean distance in a simulated genome where *var* or *pir* genes occurred at random. Differences between the actual and simulated datasets were assessed by Welch’s t-test, two-tailed.

**Additional file 4 Fig. S1.** PQSs associated with upsB-type *var* genes in Laveranian genomes. Bar plots show the number of upsB-*var-*associated PQSs as a percentage of total *var*-associated PQSs, found by *QGRS Mapper* (blue), and *G4 Hunter* (orange), in Laveranian genomes. PQSs were considered “upsB-*var*-associated” if they were found within the coding region of a upsB-type *var* gene, or if the nearest gene, within 2 kbp, was a upsB-type *var* gene.

**Additional file 5 Fig. S2.** Associations between PQSs and variantly-expressed multigene families besides *var* in Laveranian genomes. Bar plots compare the number (A) and proportion (B) of genes in non-*var* variantly-expressed multigene families that contain at least one PQS, among Laveranian species. Data for *QGRS Mapper* is shown in blue; data for *G4 Hunter* is shown in orange. Copy numbers of genes in each family were found in PlasmoDB.

**Additional file 6 Table S4.** Associations between PQSs and variantly-expressed multigene families besides *var* in Laveranian genomes. Numbers and proportions of genes in non-*var* variantly-expressed multigene families that contain at least one PQS, in Laveranian species. Copy numbers of genes in each family were found in PlasmoDB.

**Additional file 7 Fig. S3.** Conserved PQS-associated genes in *Plasmodium* species. For each species, we compiled a list PQS-associated gene IDs (found by either *QGRS Mapper* or *G4 Hunter)*, as well as their gene annotations. The table lists PQS-associated gene annotations that were common among certain groups of *Plasmodium* species.

**Additional file 8 Table S5.** GO terms analysis of PQS-associated genes for Laverania, rodent, and avian malaria parasites. Lists of PQS-associated genes for each species were analyzed for gene ontology (GO) terms in PlasmoDB. The table shows overrepresented GO terms for molecular function and biological processes for each species, as well as binning assignments that were used for the construction of the heat maps in Fig. 7.

**Additional file 9 Fig. S4.** Analysis of concordance among motifs predicted by *QGRS Mapper*, low-threshold *G4 Hunter* and experimental G4 seq in the *P. falciparum* 3D7 genome. Venn diagrams show overlaps between PQS sequences detected by *QGRS Mapper*, *G4 Hunter* and experimental G4-seq. The concordance analysis was performed with data from *G4 Hunter* at two different thresholds: the more stringent threshold of 1.7 was used in Fig. 9 B and C, whereas a threshold of 1.25 is depicted here. For G4-seq data, For G4-seq data, PQSs within 20 kb of chromosomal ends were considered telomeric. Due to the size of the G4-seq windows, it was possible for multiple PQSs to fall within the same window. Numbers in parentheses show the “full” count of PQSs detected within G4-seq windows, while numbers not in parentheses show the number of unique G4-seq windows that contained at least one PQS.

**Additional file 10 Fig. S5.** Analysis of G4-folding capacity in motifs found by *G4 Hunter* and *QGRS Mapper*. Bar plots (A, B) show fluorescent emission of the G4-specific dye thioflavin T in the presence of PQS oligonucleotides, scrambled-sequence controls, and a duplex of A/T sequence that does not form a G4 (representing background emission in the presence of DNA). ‘Relative fluorescence’ is relative to that of thioflavin T alone (no DNA). (A) shows two canonical PQSs from *P. falciparum* that were previously characterised as G4-folding via several biophysical assays [31, 37]. (B) shows three new PQSs predicted bv *G4 Hunter* in several Laveranian genomes. Data are the mean of *n* = 4 experiments conducted in technical triplicate; error bars are SD; ****, *P* < 0.0001. Table (C) shows the oligonucleotide sequences used.

**Additional file 11 Table S6.** Comparison of *QGRS Mapper* and *G4 Hunter* with experimental G4-seq data. The in silico predictions by *QGRS* Mapper and *G4 Hunter* were compared to experimental G4-seq data [29] by searching for instances where PQS start/end locations overlapped with G4-seq window locations. The table shows PQS sites and indicates whether they were found within a G4-seq window.

**Additional file 12 Table S7.** Putative G-quadruplex-forming sequences in the genomes of *Plasmodium* parasites, found by *QGRS Mapper*. Genomes were downloaded from PlasmoDB, v. 44, and searched using *QGRS Mapper* with the parameters: regex: *G*_3_*N*_(0 − 11)_*G*_3_*N*_(0 − 11)_*G*_3_*N*_(0 − 11)_*G*_3_, max length: 45, min G-group: 3, loop size: 0–11. “RC_Location Calculated” refers to the location of PQSs on the antisense strand, relative to the 5′ end of the sense strand. The “Strand (cf gene or nearest gene)” category was denoted as antisense if the PQS was on the opposite strand of its nearest gene, or sense if the PQS was on the same strand as its nearest gene. The “distance from nearest gene” was positive if the PQS was upstream of the nearest gene, negative if the PQS was downstream of the nearest gene, or equal to 0 if the PQS was in the coding sequence of the nearest gene.

**Additional file 13 Table S8.** Putative G-quadruplex-forming sequences in the genomes of *Plasmodium* parasites, found by *G4 Hunter*. Genomes were downloaded from PlasmoDB, v. 44, and searched using *G4 Hunter* with the parameters: threshold: 1.7, window size: 25, csv data: grouped. An additional search of the *P. falciparum* genome was also conducted at a lower threshold of 1.25. As per the *G4 Hunter* algorithm, PQSs on the reverse complement sequence had negative scores, and all locations were reported relative to the 5′ end of the sense strand. The “Strand (cf gene or nearest gene)” category was denoted as antisense if the PQS was on the opposite strand of its nearest gene, or sense if the PQS was on the same strand as its nearest gene. The “distance from nearest gene” was positive if the PQS was upstream of the nearest gene, negative if the PQS was downstream of the nearest gene, or equal to 0 if the PQS was in the coding sequence of the nearest gene.

## Data Availability

The original datasets supporting the conclusions of this article are available in the published literature [29] or are included within the article and its additional files. The code used in the analysis is available at https://github.com/hgage/G4s-across-Plasmodium-evolution.

## Abbreviations

G4: G-quadruplex
GO: Gene Ontology
PfEMP1: *Plasmodium falciparum* erythrocyte membrane protein 1
pir: *Plasmodium* interspersed repeat
PQS: Putative quadruplex sequence
ups: Upstream sequence
