# Supplementary figures and images for: Conserved associations between G-quadruplex-forming DNA motifs and virulence gene families in malaria parasites
Source: BMC Genomics. 2020 Mar 17;21:236. doi: 10.1186/s12864-020-6625-x (PMC7077173; doi:10.1186/s12864-020-6625-x)

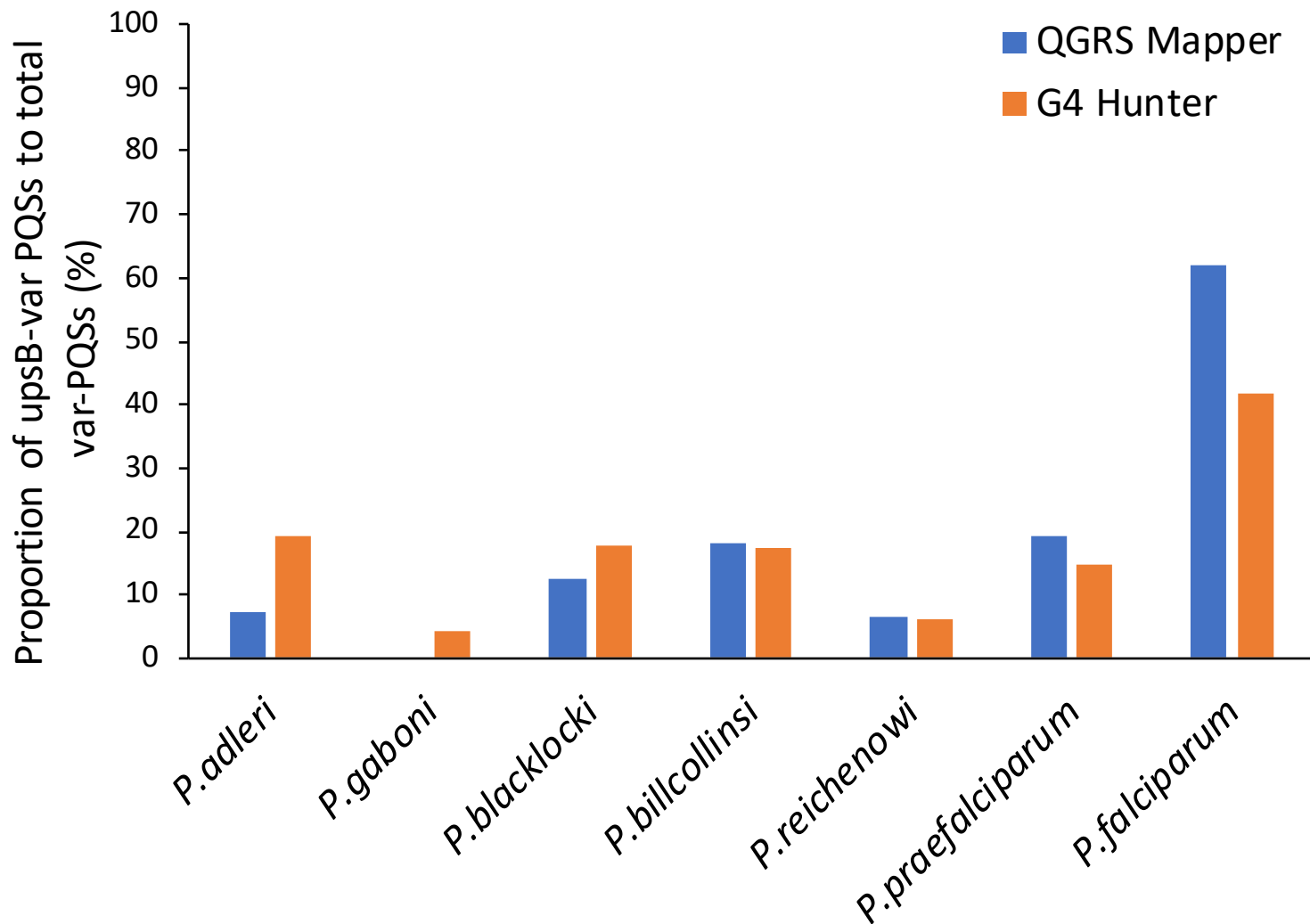

Supplement: Supplementary file 4 — Additional file 4 Fig. S1. PQSs associated with upsB-type var genes in Laveranian genomes. Bar plots show the number of upsB-var-associated PQSs as a percentage of total var-associated PQSs, found by QGRS Mapper (blue), and G4 Hunter (orange), in Laveranian genomes. PQSs were considered “upsB-var-associated” if they were found within the coding region of a upsB-type var gene, or if the nearest gene, within 2 kbp, was a upsB-type var gene. [file 12864_2020_6625_MOESM4_ESM.pdf]

**A**

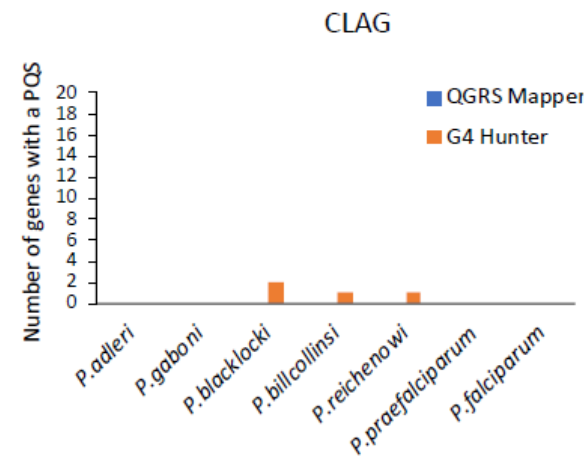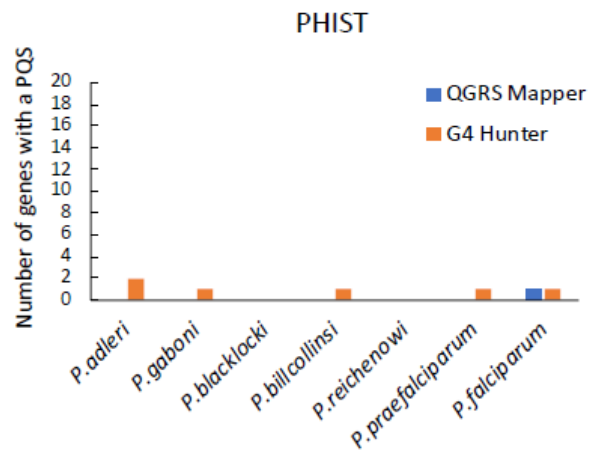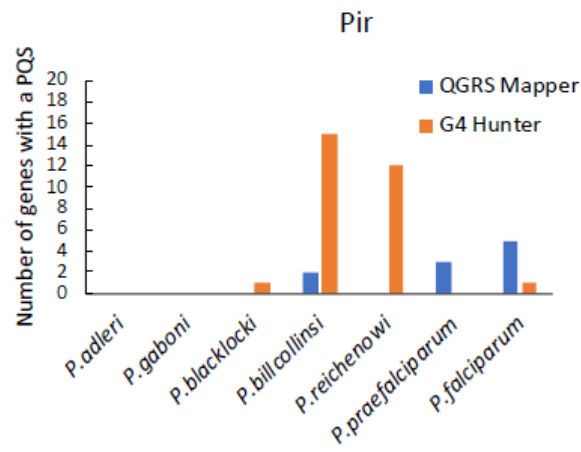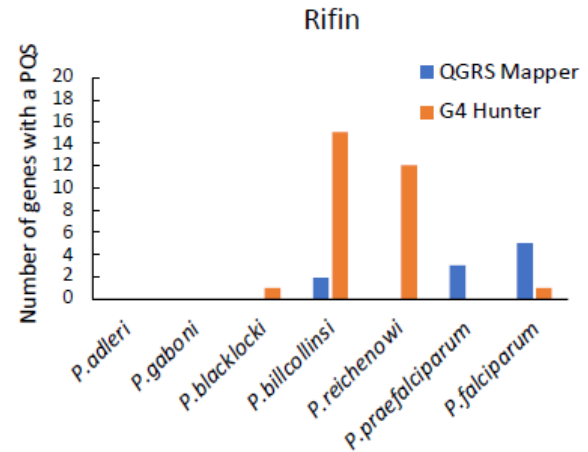

**B**

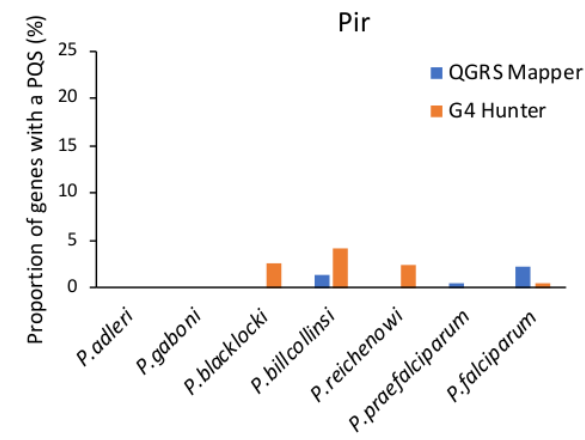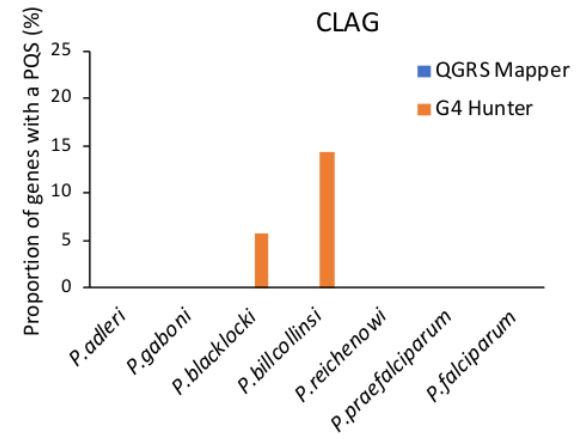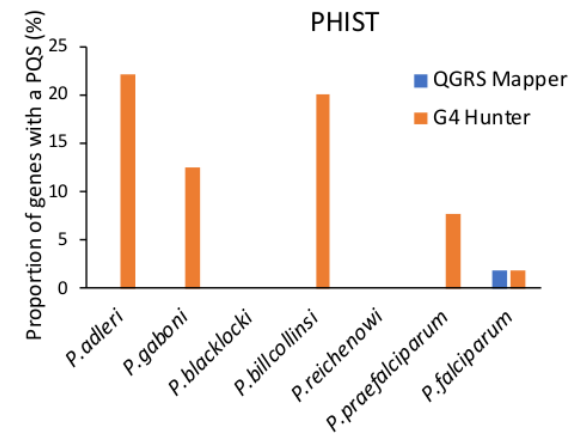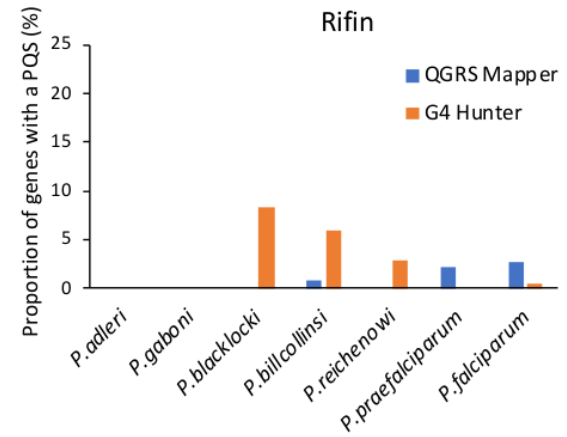

Supplement: Supplementary file 5 — Additional file 5 Fig. S2. Associations between PQSs and variantly-expressed multigene families besides var in Laveranian genomes. Bar plots compare the number (A) and proportion (B) of genes in non-var variantly-expressed multigene families that contain at least one PQS, among Laveranian species. Data for QGRS Mapper is shown in blue; data for G4 Hunter is shown in orange. Copy numbers of genes in each family were found in PlasmoDB. [file 12864_2020_6625_MOESM5_ESM.pdf]

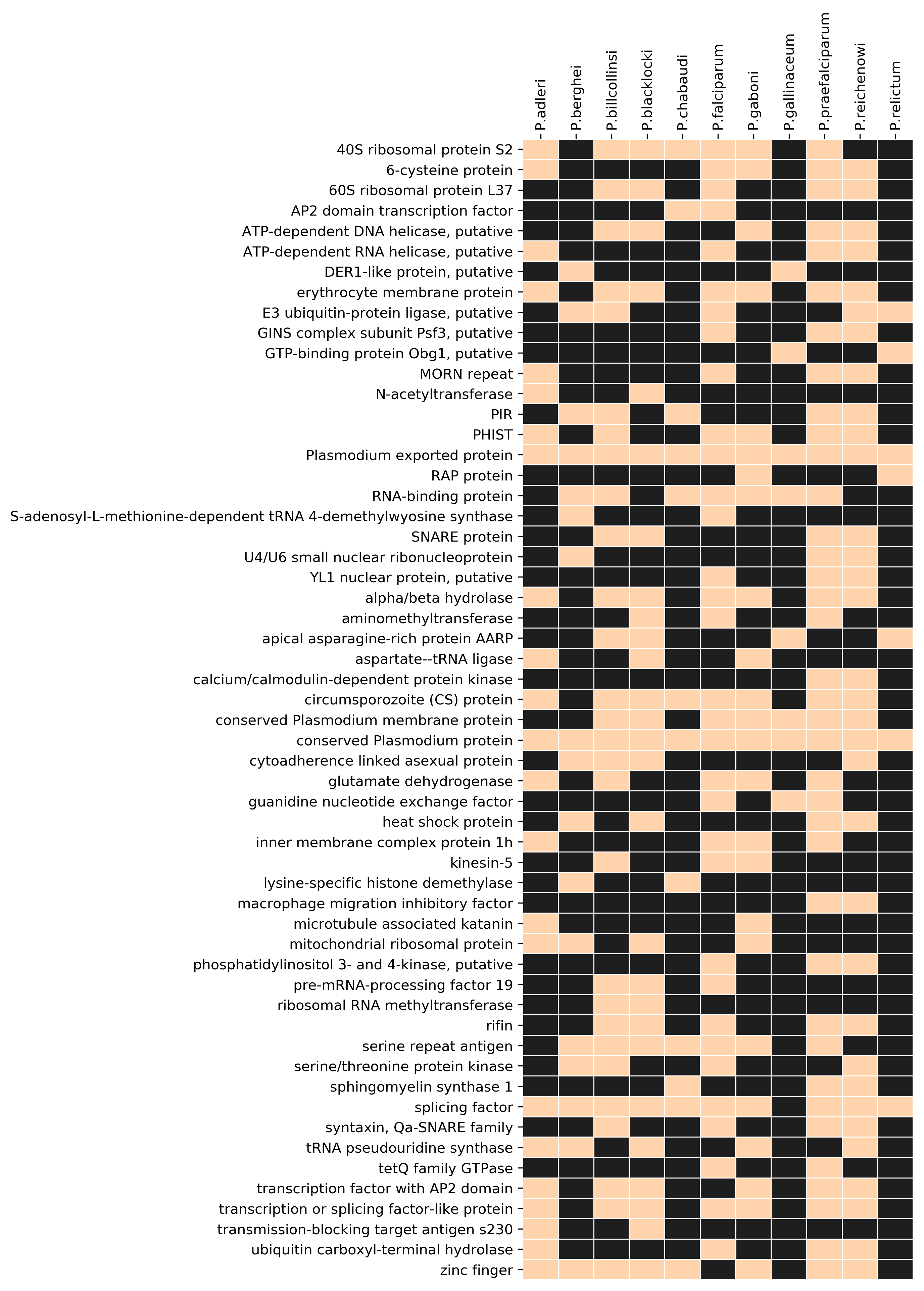

Supplement: Supplementary file 7 — Additional file 7 Fig. S3. Conserved PQS-associated genes in Plasmodium species. For each species, we compiled a list PQS-associated gene IDs (found by either QGRS Mapper or G4 Hunter), as well as their gene annotations. The table lists PQS-associated gene annotations that were common among certain groups of Plasmodium species. [file 12864_2020_6625_MOESM7_ESM.png]

**A****Telomeric**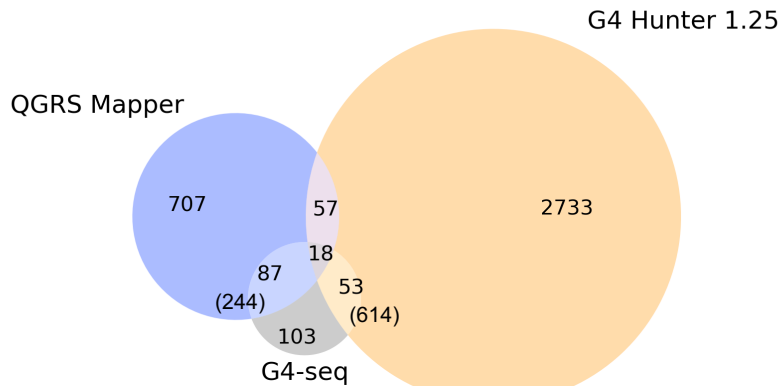**B****Non-telomeric**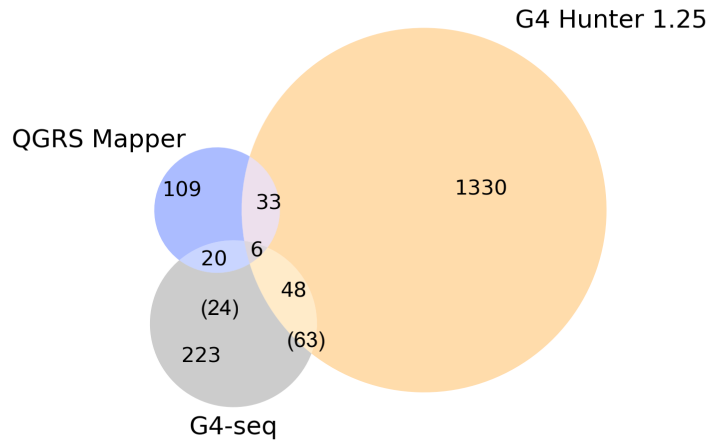

Supplement: Supplementary file 9 — Additional file 9 Fig. S4. Analysis of concordance among motifs predicted by QGRS Mapper, low-threshold G4 Hunter and experimental G4 seq in the P. falciparum 3D7 genome. Venn diagrams show overlaps between PQS sequences detected by QGRS Mapper, G4 Hunter and experimental G4-seq. The concordance analysis was performed with data from G4 Hunter at two different thresholds: the more stringent threshold of 1.7 was used in Fig. 9 B and C, whereas a threshold of 1.25 is depicted here. For G4-seq data, For G4-seq data, PQSs within 20 kb of chromosomal ends were considered telomeric. Due to the size of the G4-seq windows, it was possible for multiple PQSs to fall within the same window. Numbers in parentheses show the “full” count of PQSs detected within G4-seq windows, while numbers not in parentheses show the number of unique G4-seq windows that contained at least one PQS. [file 12864_2020_6625_MOESM9_ESM.pdf]
