# Supplementary material for: Conserved associations between G-quadruplex-forming DNA motifs and virulence gene families in malaria parasites
Source: BMC Genomics. 2020 Mar 17;21:236. doi: 10.1186/s12864-020-6625-x (PMC7077173; doi:10.1186/s12864-020-6625-x)
Supplement: Supplementary file 10 — Additional file 10 Fig. S5. Analysis of G4-folding capacity in motifs found by G4 Hunter and QGRS Mapper. Bar plots (A, B) show fluorescent emission of the G4-specific dye thioflavin T in the presence of PQS oligonucleotides, scrambled-sequence controls, and a duplex of A/T sequence that does not form a G4 (representing background emission in the presence of DNA). ‘Relative fluorescence’ is relative to that of thioflavin T alone (no DNA). (A) shows two canonical PQSs from P. falciparum that were previously characterised as G4-folding via several biophysical assays [31, 37]. (B) shows three new PQSs predicted bv G4 Hunter in several Laveranian genomes. Data are the mean of n = 4 experiments conducted in technical triplicate; error bars are SD; ****, P < 0.0001. Table (C) shows the oligonucleotide sequences used. [file 12864_2020_6625_MOESM10_ESM.pdf]

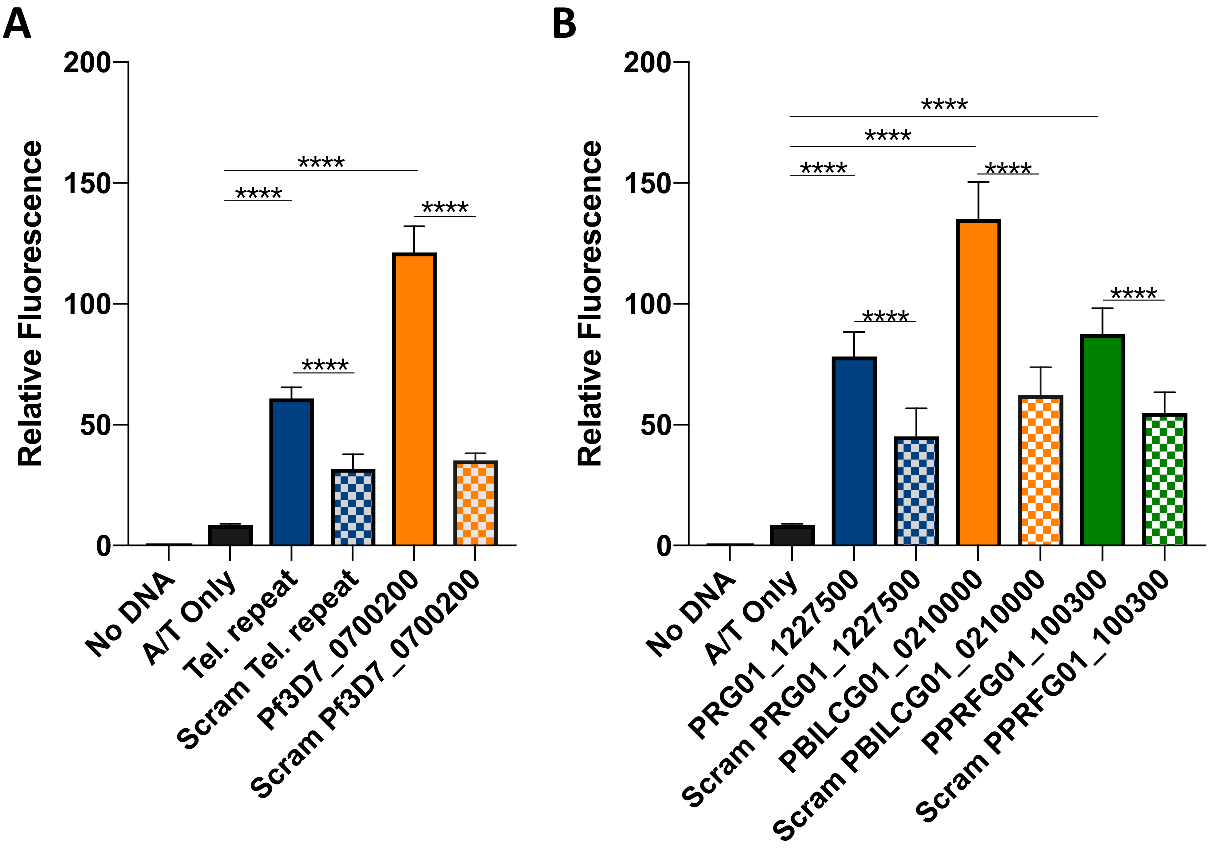

C

Oligo Name	Sequence	Species	Algorithm
AT Only	TTAAAATTTATAATTATAAATTTTAA	n/a	
Tel. repeat	GGGTTTAGGGTTTAGGGTTTAGGG	All	QGRS Mapper
Scram Tel. repeat	GTGTGTGTGTGAGTGATGTGTGAG		
Pf3D7_0700200	TTTGGGAGGGCTTGTTCCGGGAATGGGTT	*P. falciparum*	QGRS Mapper
Scram Pf3D7_0700200	GAGTGTGTGCGTCGAGTGTTAGTGTCGTG		
PBILCG01_0210000	TGGGGTTATTATTGGGGGGATGAAGG	*P. billcollinsi*<br>*P. praefalciparum (PPRFG01_0207400)*<br>*P. blacklocki (PBLACG01_0204100)*<br>*P. falciparum (PF3D7_0207400)*	G4 Hunter
Scram PBILCG01_0210000	TGTGAGTGTGAGTGTGGAGATGAGTG		
PPRFG01_100300	GAGGATGGTTATGTTGTGGGGGGGG	*P. praefalciparum*<br>*P. falciparum (PF3D7_0102200)*	G4 Hunter
Scram PPRFG01_100300	GGGAGTGTGAGTGTGTGAGTGTGGG		
PRG01_1227500	GGGGTTGGGTTAGGGTCTATGAGGGG	*P. reichenowi*<br>*P. praefalciparum (PPRFG01_1230500)*<br>*P. billcollinsi (PBILCG01_1224200)*<br>*P. falciparum (PF3D7_1224200)*	G4 Hunter & QGRS Mapper
Scram PRG01_1227500	GTGGAGTGTGTGAGAGGCGGTGTGTG		
